# Sex-Related Differences of Lipid Metabolism Induced by Triptolide: The Possible Role of the LXRα/SREBP-1 Signaling Pathway

**DOI:** 10.3389/fphar.2016.00087

**Published:** 2016-03-31

**Authors:** Zhenzhou Jiang, Xiao Huang, Shan Huang, Hongli Guo, Lu Wang, Xiaojiaoyang Li, Xin Huang, Tao Wang, Luyong Zhang, Lixin Sun

**Affiliations:** ^1^Jiangsu Key Laboratory of Drug Screening and Jiangsu Center for Pharmacodynamics Research and Evaluation, China Pharmaceutical UniversityNanjing, China; ^2^Key Laboratory of Drug Quality Control and Pharmacovigilance, Ministry of Education, China Pharmaceutical UniversityNanjing, China; ^3^State Key Laboratory of Natural Medicines, China Pharmaceutical UniversityNanjing, China; ^4^Jiangsu Key Laboratory of TCM Evaluation and Translational Research, China Pharmaceutical UniversityNanjing, China

**Keywords:** triptolide, hepatotoxicity, lipid metabolism, liver X receptor α, Sterol regulatory element-binding transcription factor 1

## Abstract

Triptolide, a diterpenoid isolated from the plant *Tripterygium wilfordii* Hook. f., exerts a unique bioactive spectrum of anti-inflammatory and anticancer activities. However, triptolide’s clinical applications are limited due to its severe toxicities. Fatty liver toxicity occurs in response to triptolide, and this toxic response significantly differs between males and females. This report investigated the pathogenesis underlying the sex-related differences in the dyslipidosis induced by triptolide in rats. Wistar rats were administered 0, 150, 300, or 450 μg triptolide/kg/day by gavage for 28 days. Ultrastructural examination revealed that more lipid droplets were present in female triptolide-treated rats than in male triptolide-treated rats. Furthermore, liver triglyceride, total bile acid and free fatty acid levels were significantly increased in female rats in the 300 and 450 μg/kg dose groups. The expression of liver X receptor α (LXRα) and its target genes, cholesterol 7α-hydroxylase (CYP7A1) and Sterol regulatory element-binding transcription factor 1(SREBP-1), increased following triptolide treatment in both male and female rats; however, the female rats were more sensitive to triptolide than the male rats. In addition, the expression of acetyl-CoA carboxylase 1(ACC1), a target gene of SREBP-1, increased in the female rats treated with 450 μg triptolide/kg/day, and ACC1 expression contributed to the sex-related differences in the triptolide-induced dysfunction of lipid metabolism. Our results demonstrate that the sex-related differences in LXR/SREBP-1-mediated regulation of gene expression in rats are responsible for the sex-related differences in lipid metabolism induced by triptolide, which likely underlie the sex-related differences in triptolide hepatotoxicity. This study will be important for predicting sex-related effects on the pharmacokinetics and toxicity of triptolide and for improving its safety.

## Introduction

Triptolide is a diterpenoid isolated from the plant *Tripterygium wilfordii* Hook. f., a member of the Celastraceae family. *Tripterygium wilfordii* Hook. f. possesses multiple pharmacological activities, including anti-inflammatory (Wang et al., 2014c), anti-fertility (Li et al., 2015), anti-cystogenesis, and anticancer activities (Li et al., 2015). However, triptolide has a narrow therapeutic window, and its clinical applications are limited due to its severe toxicities, including hepatotoxicity (Li X. et al., 2014), nephrotoxicity (Sun et al., 2013), immunotoxicity (Wang et al., 2014a), and developmental and reproductive toxicities (Ni et al., 2008; Ma et al., 2015). Among the adverse effects of triptolide, hepatotoxicity is non-negligible and has a high incidence. Our previous toxicokinetic data indicated that the hepatotoxicity induced by triptolide correlates with its highest concentration in the liver (unpublished data). Thus, it is essential to elucidate the mechanism of TP-induced hepatotoxicity.

Sex-related differences in the toxicity of triptolide have received attention in recent years. Liu et al. (2010) and Liu L. et al. (2015) reported that there are significant differences between the sexes in the toxicity of triptolide. These reports indicated that the sex-related differential expression of Cytochrome P450 3A2 (CYP3A2) in rats is responsible for sexual dimorphic metabolism of triptolide. Significant gender differences are observed in the quantity of recovered metabolites, which are likely caused by the gender-specific expression of sulfotransferases (Liu et al., 2014). We also previously reported that fatty liver is one form of toxicity caused by triptolide, and males and females exhibit significant differences in the degree of this toxicity. However, the pathogenesis underlying the sex-related differences in lipid metabolism induced by triptolide has not been elucidated and remains controversial. To date, mitochondrial respiratory chain inhibition, oxidative stress, DNA damage, and fatty acid oxidation have been proposed to be involved in triptolide-induced hepatotoxicity (Fu et al., 2011; Wang et al., 2013; Wang et al., 2014b; Liu X. et al., 2015). Although the hepatotoxicity induced by triptolide in animals and humans has been reported by many researchers, there is no reasonable explanation for the sex-related differences in lipid metabolism induced by triptolide, and it remains an issue of debate. In this study, we investigated the effects of oral triptolide on lipid metabolism in rats. The mechanisms underlying the sex-related differences in lipid metabolism induced by triptolide were examined, and the present results represent the first evidence that the LXR/SREBP-1 signaling pathway may play an important role.

## Materials and Methods

### Chemicals and Animals

Triptolide (purity > 98%, HPLC) was a gift from the Institute of Dermatology, Chinese Academy of Medical Sciences and Peking Union Medical College (Nanjing, China).

Male and female Wistar rats (180–200 g) were obtained from the Experimental Animal Center of the Academy of Military Medical Sciences. The animals were housed under identical conditions in an aseptic facility and given free access to water and food. Male and female rats were randomly assigned to the following four groups (*n* = 10/group): a control group (0.2% CMC-Na, control solution), a low-dose group (150 μg/kg/day), a middle dose group (300 μg/kg/day) and a high-dose group (450 μg/kg/day). The rats were gavaged daily with triptolide for 28 days at 5.36–16.07% of oral lethal dose 50% (2.8 mg/kg), which was determined in our previous study. The animals were monitored for the appearance of diarrhea, body weight loss, and other signs of distress. This study was carried out in strict accordance with the recommendations in the Guide for the Care and Use of Laboratory Animals of the National Institutes of Health. Protocols described were approved by China Pharmaceutical University Laboratory Animal Care and Use Committee.

After the rats were exposed to triptolide for 28 days, they were anasthetized with urethane, and blood was collected via orbital sinus bleeding for serum chemistry analysis, including the serum total cholesterol (TC), total bile acid (TBA) and low density lipoprotein-cholesterol (LDL-C) levels.

#### Transmission **E**lectron **M**icroscopy

The 1-mm^3^ thick liver tissue blocks were immediately fixed in 2.5% glutaraldehyde for 2 h at 4°C. The blocks were then treated with 1.5% OsO_4_ in 0.1 M phosphate buffer. After being washed with distilled water three times, the blocks were dehydrated and embedded in epoxy resin. Semithin sections were prepared and stained with toluidine blue. Ultrathin sections of 60 nm were stained with uranyl acetate and lead citrate and were subsequently examined with a transmission electron microscope (JEOL 1010, Japan).

### Biochemical Analysis of the Liver Tissue

The liver tissue levels of lipid metabolic parameters [triglyceride (TG) and free fatty acid (FFA) levels] were determined by a microplate reader (TECAN Safire II, Austria) using commercially available colorimetric assay kits (Nanjing Jiancheng Bioengineering Institute, China). The enzyme rate method was used to measure the TBA contents with a TBA Reagent Kit (Witman Biotech, China) using an automatic biochemical analyzer (Olympus AU2700, Japan).

### Enzyme Activity Assays

The enzymes were prepared from liver homogenates and assayed for acetyl-CoA carboxylase1 (ACC1), 3-hydroxy-3-methyl-glutaryl-CoA reductase (HMGR) and cholesterol 7-alpha-hydroxylase (CYP7A1) activity using an enzyme-linked immunosorbent assay with the respective kits (R&D, USA).

### Immunohistochemistry

Immunohistochemical staining was performed on paraffin-embedded sections (4 μm) using a microwave-based antigen retrieval technique with a ChemMate Envision kit (Dako, Denmark). The antibodies used in this study included primary antibodies against ACC1 (Millipore, USA), SREBP-1 and CYP7A1 (Santa Cruz, CA, USA). After being immunostained with secondary antibodies, the sections were developed with diaminobenzidine to produce a brown product and were counterstained with hematoxylin. The stained tissues were examined by light microscopy (Nikon E600, Japan).

### Real-Time Quantitative PCR

Total RNA was prepared from liver samples using TRIzol Reagent (Invitrogen, USA), and RNA (1 μg) was used for first-strand cDNA synthesis using RevertAid^TM^ First Strand cDNA Synthesis Kit (Fermentas, Canada). Real-time quantitative PCR was performed using gene-specific primers and SYBR Premix Ex Taq II (TaKaRa, Japan) as a fluorescent dye to detect the presence of double-stranded DNA by a real-time PCR detection system (Bio-Rad iQ5, USA). The mRNA values for each gene were normalized to those of an internal control, β-actin mRNA. The ratio of the normalized mean value for each treatment group to the vehicle control group was calculated. The primer sequences used were as follows: LXRα forward: 5′-TCC TCA GTC TGC TCC ACC-3′, reverse: 5′-TGC TCT CCG AGA TCT GGG-3′; ACC1 forward: 5′-AAG GCT ATG TGA AGG ATG-3′, reverse: 5′-CTG TCT GAA GAG GTT AGG-3′; CYP7A1 forward: 5′-GAC ACA GAA GCA TTG ACC-3′, reverse: 5′-GTA ACA GAA GGC ATA CAT CC-3′; and SREBP-1 forward: 5′-CAT CAA CAA CCA AGA CAG TG-3′, reverse: 5′-GAA GCA GGA GAA GAG AAG C-3′. The thermal cycler conditions included hold for 30 s at 95°C, followed by 40 cycles of 5 s at 95°C and 30 s at 60°C. A melting curve analysis was performed for each reaction with a 65–95°C ramp.

### Western Blot Analysis

Nuclear proteins were prepared from rat liver homogenates using Nucl-Cyto-Mem Preparation Kit (Applygen Technologies, China), and the protein concentration was determined using the bicinchoninic acid (BCA) method. The nuclear extracts were resolved on 12% SDS-poly-acryl amide gels and transferred to nitrocellulose membranes. Immunoblots were blocked overnight at 4°C with 5% BSA in TBS buffer and were incubated with primary antibodies. Immunoreactive bands were detected using a horseradish peroxidase–conjugated secondary antibody and SuperSignal Substrate (Pierce, USA). The signals were detected using a chemiluminescence detection system (Bio-Rad ChemiDoc XRS, USA).

### Statistical Analysis

The data are expressed as the means ± SD. The significance of the differences between the groups was evaluated by one-way analysis of variance (ANOVA) followed by Dunnett’s multiple comparison *post hoc* test. Statistics were performed using GraphPad Pro (GraphPad Software Inc., San Diego, CA, USA). The differences were considered significant at *p* < 0.05.

## Results

### Effects of Triptolide on the TC, TBA, and LDL-C Levels in Serum

**FIGURE 1:**
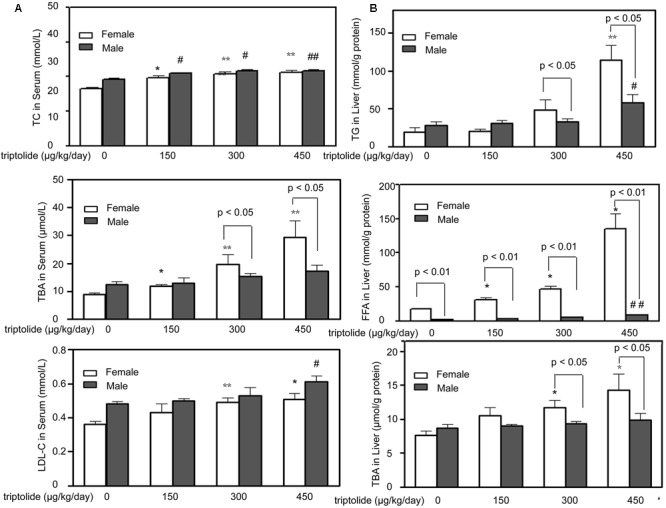
**Effects of triptolide on serum chemistry parameters and hepatic lipid accumulation in rats. (A)** The effects of triptolide on the serum levels of TC, TBA and LDL-C. Serum collected from rats treated with triptolide for 28 days was used for the analysis of the chemistry parameters. **(B)** The TG, FFA and TBA levels in the liver. All results are presented as the means ± SD for *n* = 6 animals for each group. Statistical significance relative to the vehicle control within the female group: ^∗^*p* < 0.05; ^∗∗^*p* < 0.01. Statistical significance relative to the vehicle control within male group: ^#^*p* < 0.05; ^##^*p* < 0.01. Single-line brackets indicate the difference between male and female rats. ^∗^*p* < 0.05; ^∗∗^*p* < 0.01 for respective control groups by Student’s *t*-test.

To study the effects of triptolide treatment on lipid metabolism, we first evaluated the effects of triptolide on the serum chemistry parameters of the rats. Our results indicated that the serum TC and LDL-C levels increased after triptolide treatment in both sexes and there were no statistically significant differences between male and female. The triptolide-treated female rats exhibited a significant increase in the level of TBA compared with the control group; however, this change was not observed in male rats. For female groups, the serum TBA levels of triptolide (150, 300, and 450 μg/kg /day)-exposed rats increased to approximately 33.03% (*p* < 0.05), 119.89% (*p* < 0.01), and 228.99% (*p* < 0.01) than controls, respectively (**Figure 1A**).

### Triptolide Induces Different Levels of Lipid Accumulation and Ultrastructural Changes in the Liver of Male and Female Rats

The TG, FFA and TBA levels in the liver of male and female rats are presented in **Figure 1B**. The liver TG levels of female rats in the 300 and 450 μg/kg /day dose group increased 131.29 % (*p* < 0.05) and 380.90% (*p* < 0.05) more than males, respectively. For the liver TBA levels, female rats increased 46.47% (300 μg/kg /day, *p* < 0.05) and 75.14% (450 μg/kg /day, *p* < 0.05) more than males, respectively. For liver FFA levels, the female rats increased 58.66% (*p* < 0.01), 70.14% (*p* < 0.01) and 456.58% (*p* < 0.01) higher than male rats in the 150, 300, and 450 μg/kg/day dose group, respectively. The *post hoc* analysis revealed that the triptolide-treated female rats exhibited significant increases in the TG, FFA and TBA levels compared with the control group, whereas the triptolide-treated male rats exhibited only slight changes.

**FIGURE 2:**
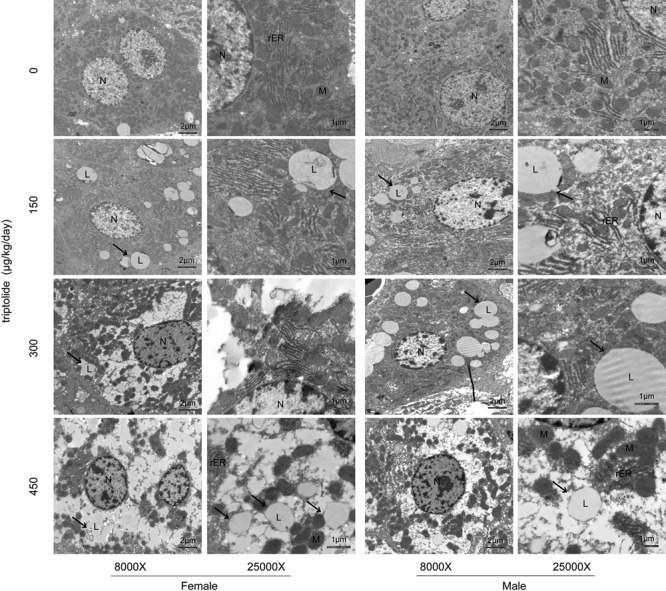
**The results of electron microscopy examination of the liver following triptolide treatment (8000X and 25000X).** Rats were administered 0, 150, 300, or 450 μg triptolide/kg/day by gavage for 28 days. Lipid droplets (L); mitochondria (M); rough endoplasmic reticulum (rER) and nuclei (N) are shown. More lipid droplets (arrow) were observed in the triptolide-treated female rats compared with the male rats.

Ultrastructural examination by transmission electron microscopy revealed that the hepatocytes of the triptolide-treated rats contained many lipid droplets, which accumulated in the cytosol. In contrast, no fat deposition was observed in the livers of the control rats. Electron microscopy also revealed dilated and degranulated rough endoplasmic reticulum (rER) in the triptolide-treated rats. In addition, the chromatin of the nucleus was massed and margined, and the mitochondria were swollen; these characteristics were not observed in the control rats. Electron microscopy revealed that the severity of the steatosis differed between female and male rats, and more lipid droplets were observed in the triptolide-treated female rats compared with the triptolide-treated male rats (**Figure 2**).

### Effects of Triptolide on the Activities of Hepatic Lipid Metabolism-Related Enzymes

**FIGURE 3:**
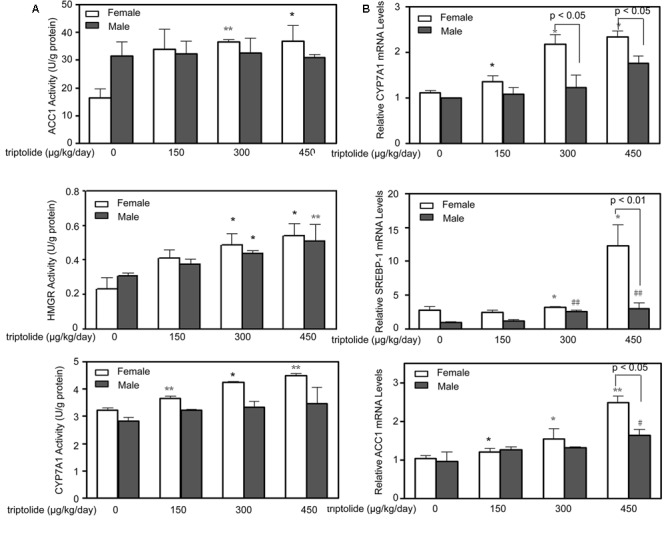
**Effects of triptolide on the activities and mRNA expression of hepatic lipid metabolism-related enzymes. (A)** The activities of ACC1, HMGR and CYP7A1 in the livers of female and male rats treated with different doses of triptolide were determined. **(B)** Real-time PCR analyses of the hepatic mRNA expression levels of CYP7A1, SREBP-1 and ACC1 were performed for both female and male rats. The gene expression levels represent the relative mRNA expression compared with the control. All results are presented as the means ± SD for *n* = 6 animals for each group. Statistical significance relative to the vehicle control within the female group: ^∗^*p* < 0.05; ^∗∗^*p* < 0.01. Statistical significance relative to the vehicle control within the male group: ^#^*p* < 0.05; ^##^*p* < 0.01. Single-line brackets indicate the difference between male and female rats.

The activities of ACC1, HMGR and CYP7A1 in the rat livers were determined. **Figure 3A** demonstrates that the hepatic ACC1, HMGR and CYP7A1 activities were all significantly (*p* < 0.05) increased in the triptolide-treated female rats. In the triptolide-treated male rats, only HMGR activity increased. The activities of HMGR of female rats in the 300 and 450 μg/kg /day dose group only showed 10.74% (*p* > 0.05) and 5.83% (*p* > 0.05) higher than males, respectively. There were no significant changes in the activities of ACC1 or CYP7A1 in the triptolide-treated male rats.

### Triptolide Affects the Expression of Key Genes and Proteins Related to Lipid Metabolism in the Liver

**FIGURE 4:**
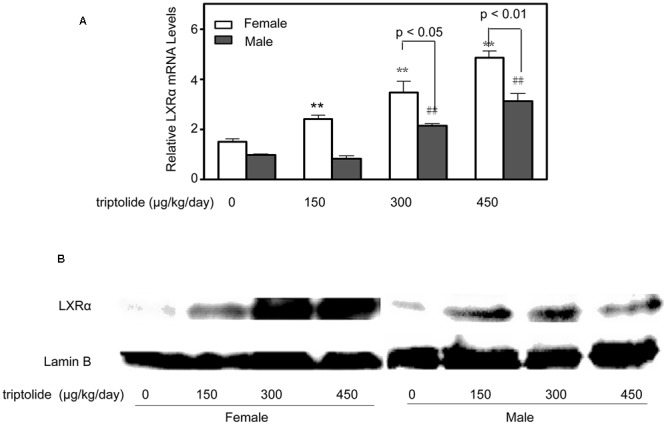
**Triptolide increases the expression of LXRα in rat livers.** Rats were administered 0, 150, 300, or 450 μg triptolide/kg/day by gavage for 28 days. **(A)** Real-time PCR analysis of the mRNA levels of LXRα was performed. Statistical significance relative to the vehicle control within the female group: ^∗∗^*p* < 0.01. Statistical significance relative to the vehicle control within the male group: ^##^*p* < 0.01. Single-line brackets indicate the difference between male and female rats. **(B)** Immunoblots for LXRα from the nuclear extracts of the liver. Lamin B was used as a loading control.

**FIGURE 5:**
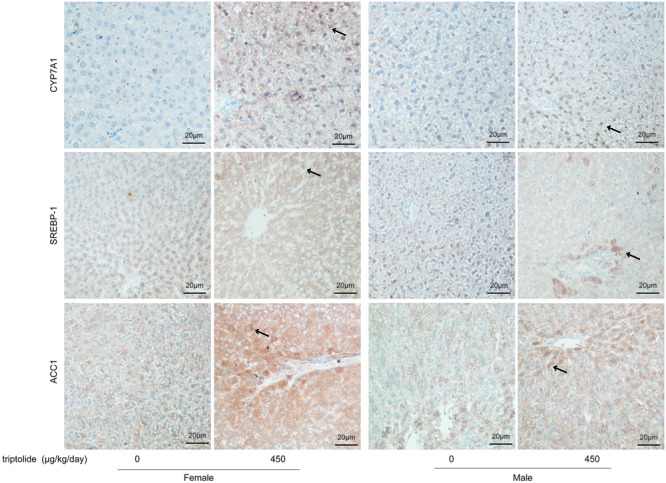
**Immunohistochemical staining of liver specimens with anti-CYP7A1, anti-SREBP-1, and anti-ACC1.** Rats were administered 0, 150, 300, or 450 μg triptolide/kg/day by gavage for 28 days. The immunostained slides were evaluated and photographed under a microscope (200X; FV-1000; Olympus, Japan). The intensity of positive brown staining (arrow) is higher in female rats than in male rats of 450 μg/kg dose group. The expression of CYP7A1, SREBP-1 and ACC1 significantly increased in the female rats in the 450 μg/kg dose group.

The effects of triptolide on the expression of the mRNA (**Figure 3B**) and proteins (**Figures 4** and **5**) that are involved in lipid homeostasis in rats were determined. Compared with the control group, the mRNA and protein expression levels of the nuclear receptor, LXRα, significantly increased in both female and male triptolide-treated rats (**Figure 4**). The expression of one of the LXRα target genes, CYP7A1, also significantly increased in the female rats in the 300 and 450 μg/kg dose groups. A significant difference in the expression of this gene was observed between males and females. SREBP-1, another gene regulated by LXRα, plays a major regulatory role in hepatic lipid biosynthesis. Real-time PCR demonstrated that SREBP-1 was more highly expressed in female rats than in male rats. SREBP-1 expression significantly increased in the female rats in the 300 and 450 μg/kg dose groups. A smaller increase in the expression of this gene was observed in male rats in the triptolide treatment groups. The lipogenic enzyme ACC1 is a target gene of SREBP-1. Real-time PCR revealed that ACC1 was also more highly expressed in female than male rats. To confirm that CYP7A1, SREBP-1 and ACC1 are involved in the sex-related differences in the triptolide-induced dysfunction of lipid metabolism, we estimated the protein level by immunohistochemical staining (**Figure 5**). The expression of CYP7A1, SREBP-1 and ACC1 significantly increased in the female rats in the 450 μg/kg dose group. A smaller increase in the expression of these genes was observed in male rats in the triptolide treatment groups.

## Discussion

Sex-related differences in human and animal toxicity have received extensive attention in recent years (Mennecozzi et al., 2015; Meeske et al., 2015). Sex-specific approaches for the prevention, diagnosis, and treatment of diseases have been examined for dyslipidemia, cardiovascular disease, metabolic syndrome, and other conditions (Pradhan, 2014; Barker-Collo et al., 2015; Brewer et al., 2015). During the past decade, the role of sex-related differences in lipid metabolism has been an intense focus of basic research and clinical treatment approaches. Our previous studies have demonstrated that triptolide exhibits sex-related differences in its toxicities (Liu et al., 2010; Tai et al., 2014). Although many reports have explored the mechanisms of hepatotoxicity induced by triptolide, no reasonable explanation of the sex-related differences in lipid metabolism has been found. In this paper, we investigated the signaling events involved in triptolide-induced dyslipidosis in the livers of female and male rats. Many genes, which form complex functional networks, collectively or cooperatively contribute to the sex-selective toxicity of drugs (Du et al., 2014; Rohrer et al., 2014). A better understanding of these links and the interplay among genes involved in drug toxicity will be of paramount importance for preventing drug-induced toxicity **(**Amacher, 2014). Therefore, in this paper, we investigated the signaling events involved in triptolide-induced dyslipidosis in the livers of female and male rats.

The liver is the major organ responsible for maintaining lipid homeostasis in the body (Hong and Tontonoz, 2014). Hepatic lipid homeostasis is tightly maintained by a balance between lipid formation (lipogenesis), catabolism (β-oxidation), and secretion (Karagianni and Talianidis, 2015). In the present study, we found that triptolide had different effects on the lipid metabolism of male and female rats. The liver TG, TBA and FFA levels increased significantly in female rats in the 300 and 450 μg/kg dose groups. Female rats were more sensitive to triptolide than males were. Electron microscopy revealed that the severity of steatosis caused by triptolide treatment differed between the female and male rats, and more lipid droplets were observed in triptolide-treated females (**Figure 2**).

LXR belong to the adopted-orphan receptors of the ligand-activated transcription factor family and form active heterodimers with the retinoid X receptor (RXR) (Lee et al., 2015). LXRα and LXRβ are key regulators of lipid and cholesterol metabolism, including bile acid and fatty acid synthesis (Schultz et al., 2000; Varin et al., 2015), and play minor or limited roles in the regulation of cholesterol synthesis and uptake in mammals. In our study, LXRα mRNA levels were increased by triptolide treatment in both male and female rats; however, the female rats were more sensitive to triptolide. Previous studies have observed noteworthy sex-related differences in LXR function in the development of atherosclerosis (Alberti et al., 2001). Transgenic overexpression of LXR in macrophages led to the upregulation of cholesterol transporters (ABCA1 and ABCG1) and a concomitant reduction in atherosclerotic lesion size only in male mice (Teupser et al., 2008). Gestational diabetes affects hypothalamic LXR expression differently in male and female offspring (Kruse et al., 2014; Korach-André and Gustafsson, 2015). The findings suggest that there are sex-related differences in the LXR-mediated regulation of gene expression. Clearly, the LXR signaling pathway leads to sex-specific modifications in lipid metabolism (Sugiyama and Agellon, 2012; Korach-André and Gustafsson, 2015). CYP7A1, a target of LXRα, catalyzes the initial step in the classical pathway of bile acid synthesis in the liver (Schaap et al., 2014). Our present results demonstrate that hepatic CYP7A1 activity and mRNA expression significantly increased in triptolide-treated female rats, with little change observed in males. Bile acids are critical for lipid absorption, particularly cholesterol, fat-soluble vitamins and, to a lesser degree, TGs (Burke et al., 2009; Zhou and Hylemon, 2014). In addition, the level of hepatic TBA was significantly increased by triptolide treatment in female rats but did not significantly change in males, paralleling the trend observed for CYP7A1 and LXR (**Figure 1**).

The SREBP family members, SREBP-1 and SREBP-2, are synthesized as membrane proteins in the ER (Xiao and Song, 2013). SREBP-1 governs fatty acid and triacylglyceride metabolism (Guo et al., 2014; Bitter et al., 2015). In contrast, SREBP-2 is deeply involved in the regulation of cholesterol metabolism (Ma et al., 2014) Reflecting such differences in biological function, the transcript for SREBP-1, which is most abundant in the liver and adrenal gland, has a shorter acidic activation domain, and the protein acts selectively to increase the mRNAs for enzymes involved in fatty acids synthesis (Soyal et al., 2015). In contrast, the ubiquitously expressed SREBP-2 transcript has a long activation domain. SREBP-2 is a potent activator of cholesterol synthesis and a weaker activator of fatty acid biosynthesis (Rice et al., 2014). Améen et al. (2004) found that the abundance of SREBP-1 mRNA was greater in female rat livers than in male rat livers. SREBP-1 is another target gene of LXRα and regulates the expression of many genes that promote fatty acid and TG synthesis, including SCD-1, ACC1, and FAS (Lee et al., 2015). Interestingly, SREBP-1 was significantly increased by triptolide treatment in the female rats in the 300 and 450 μg/kg dose groups. A smaller increase in the expression of this gene was observed in male rats in the triptolide treatment groups. In addition, the expression of ACC1, the rate-limiting enzyme for fatty acid synthesis, was markedly induced by 2.4-fold compared to the control level in female rats exposed to 450 μg triptolide/kg/day. However, no significant differences were detected between the males in the control and triptolide treatment groups. Furthermore, we detected significant increases in the levels of hepatic TGs and FFAs in triptolide-treated female rats but no significant change in these levels in triptolide-treated males.

The other SREBP isoform mentioned above, SREBP-2, is a key regulator of cholesterol homeostasis and acts as a transcription factor for HMGR (Wong et al., 2015). HMGR, which is the rate-limiting enzyme in cholesterol biosynthesis, exhibited no significant increase in rats treated with triptolide (data not shown). Furthermore, the enzyme activity of HMGR exhibited a significant increase in both male and female rats that received 300 and 450 μg triptolide/kg/day. This finding may reflect only the enzyme activity levels and not any changes in the mRNA levels, which may have been influenced by other factors.

Liu et al. (2010) and Tai et al. (2014) reported that there are significant differences between the sexes in the toxicity of triptolide. This report indicated that the sex-related differential expression of CYP3A2 in rats is mainly responsible for the sexual dimorphism of triptolide metabolism, which may result in the sex differences in triptolide toxicity. The difference in plasma and liver exposure to triptolide may be another cause of the observed sex-related differences in LXR/SREBP-1 expression. Our future work will focus on evaluation the transcriptional and post-transcriptional control of lipid homeostasis by triptolide through LXRα activation. Elucidating how the LXR/SREBP-1 pathway and sex-related factors contribute to the toxicity of triptolide will be important for predicting sex-related effects on the pharmacokinetics and toxicity of triptolide and for improving its safety.

Several research groups have reported that oxidative stress damage and mitochondrial dysfunction are important mechanisms underlying triptolide-induced liver damage (Li J. et al., 2014; Zhou et al., 2014). Our previous study indicated that the indexes of reactive oxygen species, such as MDA, SOD and XOD, were also significantly changed by triptolide in rats. Therefore, determining the indirect effect of mitochondrial dysfunction and oxidative stress on triptolide-induced dyslipidosis is an interesting issue.

## Conclusion

In conclusion, our findings indicate that triptolide can cause a significant upregulation of LXR/SREBP-1 signaling molecules in females, whereas the triptolide-treated male rats exhibited only slight changes; thus promoting an abnormal milieu for lipid metabolism which is associated with female predominance.

## Author Contributions

LZ and ZJ designed the experiments. All authors performed the experiments. XH and ZJ analyzed and discussed the data. LS, LZ, and ZJ wrote the paper. All authors contributed to the editing of the paper and to scientific discussions.

## Conflict of Interest Statement

The authors declare that the research was conducted in the absence of any commercial or financial relationships that could be construed as a potential conflict of interest.
